# Description of the design of a mixed-methods study to assess the burden and determinants of malaria transmission for tailoring of interventions (microstratification) in Ibadan and Kano metropolis

**DOI:** 10.1186/s12936-023-04684-2

**Published:** 2023-09-04

**Authors:** Ifeoma D. Ozodiegwu, Akintayo O. Ogunwale, Olabanji Surakat, Joshua O. Akinyemi, Eniola A. Bamgboye, Adeniyi F. Fagbamigbe, Musa Muhammad Bello, Al-Mukhtar Y. Adamu, Perpetua Uhomobhi, Cyril Ademu, Chukwu Okoronkwo, Monsuru Adeleke, IkeOluwapo O. Ajayi

**Affiliations:** 1 Department of Preventive Medicine and Institute for Global Health, Northwestern University, Chicago, IL USA; 2 Epidemiology and Biostatistics Research Unit, Institute for Advanced Medical Research and Training (IAMRAT), College of Medicine, University of Ibadan, Ibadan, Oyo Nigeria; 3 Department of Public Health, College of Health Sciences, Bowen University, Iwo, Osun Nigeria; 4 Department of Zoology, Osun State University, Osogbo, Osun Nigeria; 5 Department of Epidemiology and Medical Statistics, Faculty of Public Health, College of Medicine, University of Ibadan, Ibadan, Oyo Nigeria; 6 Department of Community Medicine, Bayero University, Kano, Nigeria; 7 Department of Medical Microbiology and Parasitology, Bayero University, Kano, Nigeria; 8 National Malaria Elimination Programme, Abuja, Nigeria

**Keywords:** Malaria, Urban areas, Nigeria, Microstratification, Intervention tailoring, Epidemiology, Entomology, Mixed-methods, Qualitative research, Longitudinal surveys

## Abstract

**Background:**

Rapid urbanization in Nigerian cities may lead to localized variations in malaria transmission, particularly with a higher burden in informal settlements and slums. However, there is a lack of available data to quantify the variations in transmission risk at the city level and inform the selection of appropriate interventions. To bridge this gap, field studies will be undertaken in Ibadan and Kano, two major Nigerian cities. These studies will involve a blend of cross-sectional and longitudinal epidemiological research, coupled with longitudinal entomological studies. The primary objective is to gain insights into the variation of malaria risk at the smallest administrative units, known as wards, within these cities.

**Methods/results:**

The findings will contribute to the tailoring of interventions as part of Nigeria’s National Malaria Strategic Plan. The study design incorporates a combination of model-based clustering and on-site visits for ground-truthing, enabling the identification of environmental archetypes at the ward-level to establish the study’s framework. Furthermore, community participatory approaches will be utilized to refine study instruments and sampling strategies. The data gathered through cross-sectional and longitudinal studies will contribute to an enhanced understanding of malaria risk in the metropolises of Kano and Ibadan.

**Conclusions:**

This paper outlines pioneering field study methods aimed at collecting data to inform the tailoring of malaria interventions in urban settings. The integration of multiple study types will provide valuable data for mapping malaria risk and comprehending the underlying determinants. Given the importance of location-specific data for microstratification, this study presents a systematic process and provides adaptable tools that can be employed in cities with limited data availability**.**

**Supplementary Information:**

The online version contains supplementary material available at 10.1186/s12936-023-04684-2.

## Background

Nigeria, in addition to being the greatest contributor to the global malaria burden, is rapidly urbanizing with the attendant challenges of overcrowding and environmental degradation, leading to growing concerns that malaria transmission may increase substantially in cities [1, 2]. Presently, a little over half of Nigerians reside in urban centres, and it is projected that this number will rise to 70% in 2050 [3]. Infrastructural elements due to urban development, such as a high-quality housing, are expected to reduce malaria transmission [4]. However, non-uniform infrastructure planning and provision within city neighbourhoods is resulting in the expansion of informal settlements, slums, and urban farms with suitable habitats for vector breeding [5, 6]. In addition, the discovery of *Anopheles stephensi*, a malaria vector adapted to urban areas, in Nigeria may intensify transmission risk for urban residents [1, 7, 8]. Neighbourhood-level differences in environmental suitability for mosquito breeding in cities will likely lead to small-scale geographic variation in malaria burden and these variations need to be accounted for during intervention planning.

Recognition of the implications of the spatial variation in malaria risk underlies the High Burden to High Impact (HBHI) response launched by the World Health Organization (WHO) in 2018 to reignite the pace towards achieving the Global Technical Strategy (GTS) targets of reducing malaria cases and deaths by 75 and 90% in 2025 and 2030 relative to 2015 levels [9, 10]. High-burden malaria countries like Nigeria, where declines in malaria burden have stagnated in recent years, constitute a priority for HBHI-related activities. The second element of the HBHI response calls for high-burden countries to transition from a ‘one-size-fits-all’ approach to a more targeted response to enable the deployment of the most effective malaria control tools to areas where they will have maximum impact. The Nigerian Malaria Elimination Programme (NMEP) heeded the call for a more targeted response to malaria control and, in the 2021–2025 National Malaria Strategic Plan, interventions were tailored for each Local Government Area (LGA) [11]. Mathematical models supported the selection of optimal intervention mixes for each LGA [11, 12]. Similarly, cities need intervention tailoring, particularly targeting informal settlements, slums and neighbourhoods situated close to farms, areas where residents may be at higher malaria risk as compared to planned settlements with high-quality housing and improved environmental conditions. The WHO has recommendations for targeting malaria interventions to individual geographic areas considering local epidemiology, ecology, health system and socio-economic characteristics and these can be used to inform intervention decisions [13]. Selected intervention scenarios per locality can then be evaluated with mathematical models to understand their impact and inform the development of city-level intervention plans within the national malaria strategic plans. Implementing this strategy, however, requires data that is currently lacking.

City-level variations in malaria burden and risk factors are not well-understood in Nigeria. National surveys do not have sufficient sample sizes necessary for capturing intra-urban malaria risk. While routine clinical malaria data encompasses malaria incidence in individuals living in cities, it is biased towards those that use public health facilities [14]. Socio-economic data, human behavior, information on place of residence and travel histories and frequency are typically not collected routinely; and routine data often lacks a well-defined catchment population [15]. These shortcomings of clinical malaria data preclude its use in understanding spatial distribution of malaria cases, deaths, and risk factors in cities. In addition, data from entomological surveys are usually not specific to urban geographies [16]. The research literature also provides limited information on the burden and drivers of malaria risk in cities [17–21]. These data and information gaps highlight the need for additional studies that elucidate the burden and drivers of malaria risk in urban Nigeria to inform city-level targeting of interventions.

### Study objectives

In collaboration with the NMEP, the study team will conduct human and entomological studies in Ibadan and Kano to better understand how to tailor malaria interventions in urban areas. The corresponding data will be used to construct a mathematical model of urban malaria transmission for Ibadan and Kano, which will inform Nigeria’s 2026–2030 National Malaria Strategic Plans. These cities were selected due to their high malaria burden at the state level and the potential cost savings for the NMEP that would arise from reducing malaria transmission. The study employs mixed methods approaches, with the overarching aims being to evaluate the following:

1. Ward-level prevalence of malaria,
2. Malaria seasonality,
3. Risk factors for malaria prevalence and incidence, and
4. Risk of local transmission in formal, informal settlements and slums as assessed by entomological indicators.

**Fig. 1:**
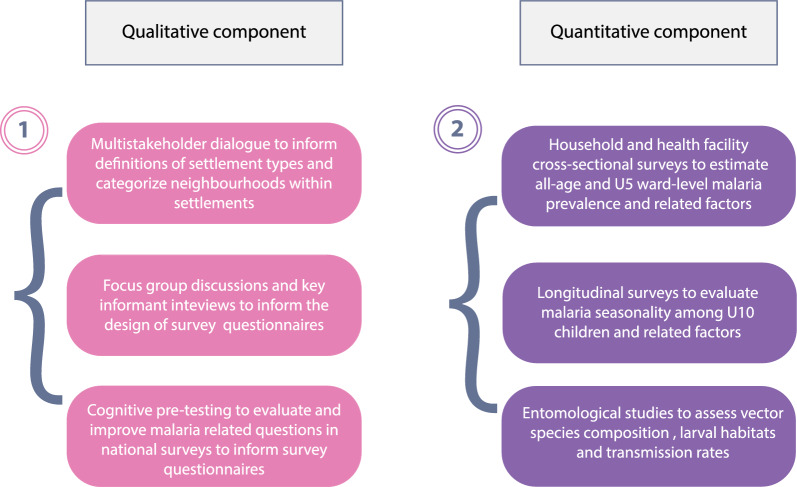
Study approach, types, and objectives. U5 is an abbreviation for children under the age of 5 years and U10 means children under the age of 10 years

Field studies will commence with formative assessments to define formal and informal settlements and slums in the study wards. These assessments will also inform survey development and study planning for both the cross-sectional and longitudinal studies. Subsequently, cross-sectional studies conducted at households and health facilities will yield data for estimating ward-level malaria prevalence and identifying key risk factors. Longitudinal studies will help estimate malaria seasonality and identify malaria risk factors for both formal and informal settlements, and slums. Entomological studies will contribute to understanding of local transmission in formal, informal settlements, and slums by yielding data on vector species composition, larval habitats, and transmission rates (Fig. 1).

The data from these field studies will be used to calibrate a ward-level mathematical model that projects the impact of different intervention scenarios on malaria burden indicators for individual wards in the Kano and Ibadan metro areas. Through this analysis, the NMEP will gain comprehensive insight into the consequences of various intervention strategies, including the impact of excluding city wards with a low transmission burden from insecticide treated nets mass campaigns. The outputs of mathematical modelling will assist the NMEP in identifying optimal strategies to achieve malaria control and elimination goals.

## Methods/results

### Description of study location and study ward selection process for the prevalence, longitudinal and entomological surveys

**Fig. 2:**
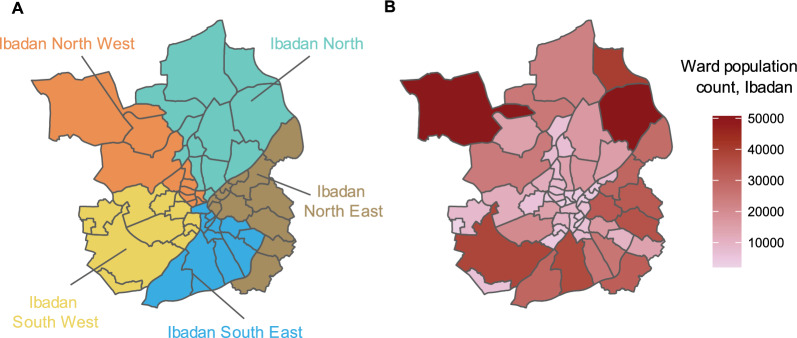
Administrative subdivisions of Ibadan metro. **A** Ward boundaries are colored by their corresponding LGAs, **B** ward population count

**Fig. 3:**
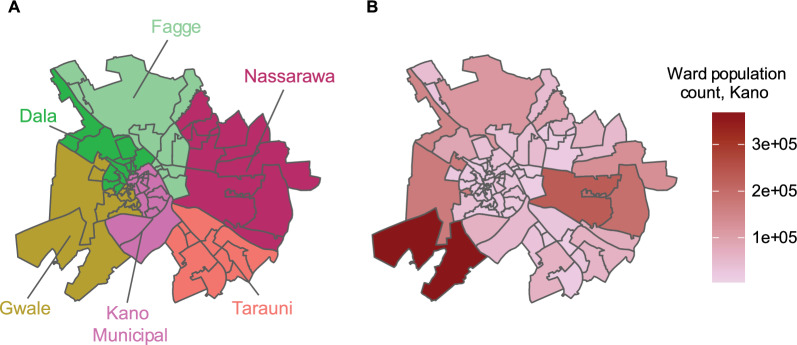
Administrative subdivisions of Kano metro. **A** Ward boundaries are colored by their LGA, **B** wards in Kano population count

Ibadan and Kano metropolis are the study locations. The Ibadan metro area is in Oyo state and is divided administratively into five LGAs, further subdivided into 59 wards (Fig. 2A). The population sizes of the wards in Ibadan metro are presented in Fig. 2B. The mean population size across all 59 wards in Ibadan is 15,408 persons (Standard Deviation (SD): 12,747) [22]. The mean population density for Ibadan is 12,665 individuals per square kilometre (SD: 5802) [23]. The Kano metro area is in Kano state and consists of six LGAs, which are subdivided administratively into 66 wards (Fig. 3A). The population sizes of the ward in Kano are depicted in Fig. 3B. The mean population size across all 66 wards in Kano is 47,497 persons (SD: 58,981) [22]. The mean population density in Kano is 23,401 individuals per square kilometre (SD: 17,313) [23].

#### Overview of the study ward selection process

**Fig. 4:**
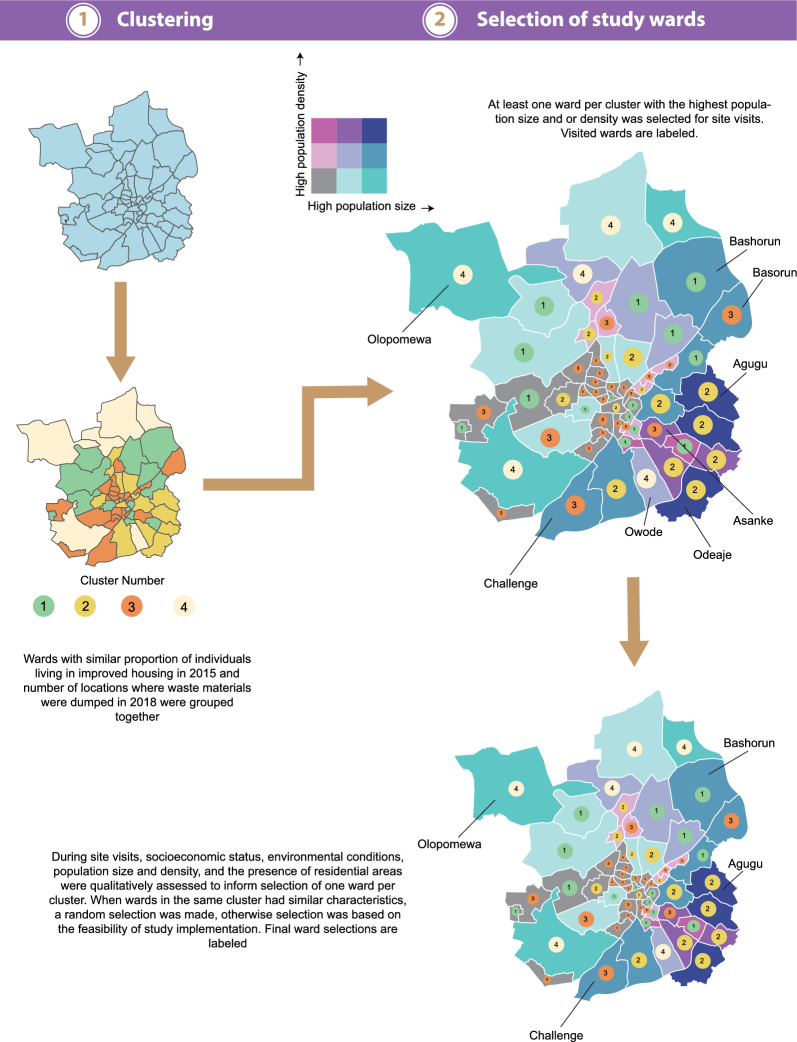
Overview of methods used to select study locations in Ibadan metropolis for the household, longitudinal and entomological surveys. Wards visited for ground truthing, and final ward selections are labeled. Similar methods were used for ward selection in Kano metropolis

Considering the significant number of wards within the LGAs of Ibadan and Kano metropolis, a methodology was developed to identify a representative subset of wards. This selected subset would allow extrapolating prevalence, seasonality, and entomological data to unsampled wards. To accomplish this, a model-based clustering approach was employed to categorize the wards in Ibadan and Kano metropolis based on their shared characteristics. Subsequently, site visits were carried out for at least one ward per cluster, prioritizing those with the highest population size or density. These visits aimed to validate the clustering outcomes and inform the final ward selection process. Figure 4 illustrates the ward selection process specifically for Ibadan, while a corresponding figure for Kano is shown in Additional file 1: Figure S1.

#### Clustering

Clustering methods offer a valuable means of identifying geographical areas that share similar characteristics. In this study, clustering techniques were employed to identify wards displaying comparable transmission patterns. To achieve this, model-based clustering with variable selection was utilized as an empirical approach to classify wards into distinct clusters [24]. Since direct measures of malaria transmission were unavailable, environmental characteristics were used as a proxy. Table 1 provides an overview of the variables considered for clustering wards in both the Ibadan and Kano metropolises, as well as the final variables utilized for clustering after variable selection. Notably, data pertaining to the number of locations where waste materials were disposed of in 2018 were exclusively used in the Ibadan clustering model due to its unavailability for Kano.

**Table 1:** Model-based clustering inputs

Metro area	Variables considered for clustering	Variable used for clustering after variable selection
Ibadan and Kano	Population density [27] Enhanced vegetation index [29] Settlement type classification, classification (received through email communication from Bill and Melinda Gates Foundation Geographic Information Systems Team), Proportion of individuals living in improved housing in 2015 [30]	Proportion of individuals living in improved housing in 2015
Ibadan alone	Number of locations where waste materials were dumped in 2018 [31]	Number of locations where waste materials were dumped in 2018 [31]

After variable selection, two variables—data on the proportion of individuals living in improved housing in 2015 and the number of locations where waste materials were dumped in 2018—were used to group wards in the Ibadan metro area into four clusters. Similarly, wards in the Kano metro area were aggregated into five clusters using data on the proportion of individuals living in improved housing in 2015. The selection of cluster partitions was based on Bayesian Information Criteria (BIC) values; those with the lowest BIC values were selected. Each variable selected as input in the clustering procedure was informed by the research literature and availability of representative geospatial data. For instance, in their 2017 publication, Kabaria and colleagues found that population density and enhanced vegetation index were important predictors of malaria risk [25]. Housing quality was among the key factors associated with malaria transmission, as identified by Silva and Marshall [26]. Additionally, Nasir et al*.* [27] found a higher prevalence of malaria among individuals living close to dumpsites compared to those who lived away from dumpsites.

Following the clustering procedure, one to three potential study locations with either the largest population size and/or population density per cluster were selected for site visits. These visits helped inform decisions on the final selection of one study location per cluster (Table 2). If one ward had both the highest population size and density, the ward with the next highest population size was chosen. Population size and density were chosen as the selection criteria for study wards based on the assumption that selected areas would comprise individuals of different socio-economic statuses. This diversity is expected to result in the generation of representative estimates of transmission within their cluster partition.

**Table 2:** Potential and final study wards

Cluster number	Ibadan metro area	Perceptions of ward characteristics per cluster in Ibadan	Kano metro area	Perceptions of ward characteristics per cluster in Kano
1	Owode, **Olopomewa**	Olopomewa and Owode exhibited significant differences. Olopomewa had tarred roads and high-quality housing. Roads in Owode were untarred and had lower-quality housing	**Dorayi**, Goron Dutse	Both Dorayi and Goron Dutse were densely populated and had a mix of low- and high-quality housing. Both wards appeared to be undergoing redevelopments
2	**Challenge,** Basorun, Asanke	Environmental characteristics were roughly similar in Challenge, Basorun and Asanke. Roads were mostly untarred and housing infrastructure was aged	**Fagge D2**, Kwachiri	Fagge D2 and Kwachiri were dissimilar. Kwachiri was described as a commercial hub with very few residential houses. Fagge D2 was a densely populated residential ward with medium to poor quality housing
3	Ode Aje, **Agugu**	Residents’ socio-economic status and environmental characteristics in Ode Aje and Agugu were observed as roughly similar. Residents were perceived to be of low-income, wards were densely populated, and roads were untarred	**Tudun Wazurchi,** Sani Mainagge	Tudun Wazruchi and Sani Mainagge were perceived to be dissimilar. Housing quality was of poor to medium quality in Tudun Wazurchi and of higher quality in Sani Mainagge
4	**Bashorun**	Only one ward was visited in this cluster. Bashorun had good road network and the socio-economic status of residents was perceived to be high	**Gobirawa**, Kofar Ruwa	Gobirawa was found to be densely populated ward with medium to low quality housing and untarred street roads. Kofar Ruwa was a commercial hub with few residential areas
5			**Zango**, Shahuchi	Both Zango and Shahuchi have varied housing quality ranging from high to poor quality. Zango appeared to have more areas where gutters were filled with rubbish

Final study wards are in bold

#### Site visits

Considering funding limitations, only one ward could be selected per cluster. Therefore, the study team conducted site visits to confirm the clustering results and inform final ward selections by assessing whether selected wards had residential areas. The team also qualitatively assessed and compared location characteristics within clusters where two or more potential study wards were visited. Eight potential study wards (Fig. 4 and Table 2) were visited in the Ibadan metro area and ten wards were visited in the Kano metro area (refer to Additional file 1: Figure S1). The study team captured pictures of residential houses and roads in each area, and qualitatively assessed and compared socio-economic status, environmental conditions, and population size and density for wards in the same cluster.

Reports from the site visits in the Ibadan metro area, for instance, suggested that wards in clusters 2 exhibited similarities in housing quality, road infrastructure, and neighbourhood conditions, while wards grouped into cluster 1 in the Ibadan metro area showed significant differences. To elaborate, most neighbourhoods in Olopemewa had tarred roads in contrast to Owode (Table 2). Regarding Kano, clustering methods captured similarities in housing quality in wards in cluster 1, but not in cluster 3. Housing infrastructure in Dorayi and Goron Dutse showed similarities, while in Tudun Wazurchi, the quality was lower than that in Sani Mainagge. Additionally, individuals residing in Tudun Wazuruchi were regarded as having a low-income status compared to those in Sani Mainagge. Considering the varying performance of the clustering methods for different clusters, decisions on study wards in clusters with dissimilar housing and environmental conditions relied on the field team’s opinion on the ease of study implementation. The selected study wards in Ibadan were four: Olopomewa; Challenge; Agugu; and Bashorun, while in Kano metro area they were five: Dorayi; Fagge D2; Tudun Wazurchi; Gobirawa; and Zango (Table 2). The final selected study locations represent a diversity of environmental and living conditions in both Ibadan and Kano metropolis; detailed site visit notes and pictures for each ward are provided in Additional file 1: Table S1 and Figures S2–19).

### Formative research methods

#### Multi-stakeholders’ Dialogues (MSDs) for defining formal and Informal settlements and slums and categorizing neighbourhoods to inform sampling design

The commencement of fieldwork will involve multi-stakeholders’ dialogues (MSDs) aimed at defining formal and informal settlements, including slums. These discussions will help categorize neighbourhoods based on their settlement types, subsequently informing the sampling design within selected wards. The MSDs are structured processes used to bring stakeholders together to develop a shared understanding of issues, evidence and plans of action [28]. One MSD will be conducted per city. The MSDs will use a participatory community mapping approach. During a roundtable discussion, participants will reach a consensus on the characteristics of different settlement types (See Additional file 1: pages 17–18 for guide). Each participant will be provided with a form and asked to share their general impressions of the study wards, as well as record the number of communities/neighbourhoods classified as formal settlements, informal settlements, or slums. Subsequently, participants familiar with or residing in each of the study wards will be asked to sketch a community map with detailed descriptions of various settlement types and to present the final product to the full group of participants for feedback. To facilitate the map sketching process, participants will be provided with a validated map of each ward showing major landmarks and streets.

In Ibadan and Kano, respectively, a total of 10 participants will be recruited for the MSDs. Participants will include a Town Planner or a Quantity Surveyor from the State Ministry of Lands and Housing, a Building/Structural Engineer from the State Ministry of Works, a Surveyor from the Office of the Surveyor-General, a Statistician/Demographer or Population Expert from the State Bureau of Statistics and an academic with expertise in Urban Planning or Geography. The MSDs will also include two stakeholders who work as Estate Managers/Agents, two community members residing in formal and informal settlements, and one intra-city experienced commercial taxicab or shuttle driver familiar with various areas within the metropolis. The recruitment of pertinent stakeholders for the MSDs will be facilitated using purposive and snowballing procedures. Community gatekeepers and heads or senior officials in various relevant ministries and institutions will be consulted and engaged for the purpose of recruiting distinguished and well-informed individuals for the MSDs. In addition to the 10 participants that will be involved in each of the MSDs, a stakeholder from the state level/office of NMEP will be involved in the dialogue as a non-participant observer. The Additional file 1: Page 16 includes a matrix showing details of the description of the participant types and how each participant will be sampled, the study guide and a sample of the informed consent form for the MSDs. Each MSD will be recorded and facilitated by a rapporteur and two moderators (facilitators) who possess expertise in qualitative research and community engagement.

#### Focus Group Discussions and Key Informant Interviews to inform the design of human survey questions

In both the Ibadan and Kano metro areas, Focus Group Discussions (FGDs) and Key Informant Interviews (KIIs) will be conducted among participants purposively selected from diverse communities, encompassing formal and informal settlements as well as slums. The designations of formal, informal settlements, and slums will be informed by the outcomes of the MSDs. Utilizing guides comprising of open-ended questions and prompts (See Additional file 1: pages 21–53), information will be gathered to guide the design of the quantitative study. The FGDs and KIIs will delve into the following topics: (1) community management of suspected malaria infections, (2) health-seeking behaviours of community members concerning malaria, (3) utilization of malaria medications for treating past suspected or confirmed malaria episodes, along with optimal strategies for assisting study participants in recalling the medications used, and (4) approaches employed by community members to safeguard themselves against malaria. As demonstrated in Table 3, the Focus Group Discussions (FGDs) will encompass community members, particularly caregivers of children under the age of five, as well as adult male and female groups. For each settlement type (formal settlements, informal settlements, and slums), FGDs will be meticulously organized, ensuring representation across different categories within the target population. Uniform discussion guides will be employed across all study groups. Each FGD group will comprise of 8–12 participants, and a minimum of nine FGDs will be conducted in both Ibadan and Kano. Additionally, two FGDs will be conducted with male and female adults who also serve as caregivers of under-fives; this will serve to pre-test the study instruments, eliminating the need for further pretesting among caregivers of under-fives. Data collection will persist until a point of saturation is reached. Participants of each FGD will be purposively recruited, with the support of community gatekeepers or community liaison officers, identified through a community engagement process.

**Table 3:** Anticipated number of focus groups per city and description of potential participants and purpose of each discussion

Recruitment target population	Anticipated number of FGDs	Purpose
Male and female adults	2 (one FGD for males and females, respectively)	Pretest study instruments
Caregivers of under-fives	3 (one FGD per settlement type)	Obtain group perspectives
Adult male	3 (one FGD per settlement type)	Obtain group perspectives
Adult female	3 (one FGD per settlement type)	Obtain group perspectives
	Total number of FGDs per city: 11	

The KIIs will center on stakeholders from the community, formal health sector, and informal health sector across the diverse settlement types in Ibadan and Kano. Key informants will be purposively selected from various settlement types within the study sites. Separate interview guides will be employed for the three groups of interviewees: community stakeholders, health workers in formal healthcare sectors, and healthcare providers in informal sectors. While distinct for each category, these KII guides will share a common focus and cover several shared topics. A minimum of twenty KIIs will be conducted at each study location (see Table 4 for details). To ensure the effectiveness of the guides, an additional two (KIIs) will be carried out for pretesting with workers from both the formal and informal health sectors. Given that health sector workers also have a role as community stakeholders, a separate pretest among community stakeholders was deemed unnecessary.

**Table 4:** Description of key informant interview types, recruitment targets and proposed number of interviews per city

Key informant interview (KII) target stakeholder	Recruitment target population	Anticipated number of KIIs	Purpose
Health sector stakeholders	Formal health worker and informal health worker	2 KIIs	To pretest study instruments
Community stakeholders	Community/opinion leaders, traditional leaders (one community leader from each of the three types of settlements)	4 KIIs	To obtain key stakeholders’ perspectives
Formal health sector stakeholders	Heads of primary health care centres, Primary Health care (PHC) coordinators, LGA medical officers, LGA Roll-back Malaria Focal Persons, Public and private sector pharmacists and doctors, and State Malaria Programme Officers	10 KIIs	To obtain key stakeholders’ perspectives
Informal health sector stakeholders	Patent Medicine Vendors, drug peddlers/hawkers, traditional/herbal healers (two separate KIIs from each of the typologies of informal health workers across the three different settlement types)	6 KIIs	To obtain key stakeholders’ perspectives
		Total number of KIIs per city: 22	

The final count of conducted Focus Group Discussions (FGDs) and KIIs will be determined based on our team’s assessment of data saturation.

Key Informant Interviews (KIIs) and Focus Group Discussions (FGDs) will be skillfully conducted by a dedicated team, featuring two trained Research Assistants (RAs) per session. Each team will consist of a moderator and a recorder/note-taker, and their endeavors will be under the supervision of field supervisors, a quality assurance manager, and a social scientist/qualitative research expert from our research team. In total, a team of ten RAs and two supervisors will be carefully selected to partake in a comprehensive 2-day training session at each site. The chosen RAs will be university graduates possessing experience in qualitative research. Additionally, they will exhibit fluency in English, Yoruba (for Ibadan), and Hausa (for Kano). Gender sensitivity will be thoughtfully integrated into the selection process.

The FGDs and KIIs will take place in settings that prioritize participant privacy, ensuring a comfortable atmosphere for sharing. Audio recordings will be captured for all sessions. Anticipated timeframes for KIIs range from approximately 25 to 45 min per session, while each FGD is projected to last around 40 to 60 min.

#### Cognitive pretesting of questions from the Nigerian Demographic and Health Surveys to inform the design of human survey questions

The Demographic and Health Survey questionnaire consists of validated questions related to malaria illness, health-seeking behaviour, and financial status, which could be valuable for the study. To assess its utility with the study population, the recall and understanding of relevant questions will be assessed through cognitive pretesting among mothers of children under the age of five (see Additional file 1: pages 56–61 for the guide). Cognitive pretesting, also referred to as Cognitive Interview (CI), will enable the study team to generate recommendations for enhancing the evaluated questions in the quantitative surveys.

At least four caregivers of children under the age of five will be purposefully selected from formal and informal settlements, and slums in the Ibadan and Kano metro areas for the CIs. Each CI session will be conducted with the assistance of a moderator and a note-taker. The conduct of each CI is anticipated to take about 20–25 min. The CIs will be conducted within the same timeline as the KIIs and FGDs.

### Survey research methods

The commencement of the cross-sectional and longitudinal studies is scheduled to follow the culmination of the formative research phase. The insights gathered from the formative research will play a crucial role in refining both the survey instruments and the sampling strategies for the cross-sectional and longitudinal studies. The following section outlines the preliminary research plans.

### Cross-sectional studies

#### Household surveys

To evaluate the prevalence of malaria at the ward level among all age groups, including children under the age of five, and associated factors such as socio-economic status, behavior, intervention coverage, and mobility, household-based surveys will be conducted in study wards during the wet season (June–August in Ibadan, August–October in Kano) and dry season (January–March in both Ibadan and Kano) among individuals of all ages, including pregnant women, from different settlement types. An equal number of households will be sampled during both the dry and wet seasons. The survey instruments are provided in Additional file 1: pages 62–78 and will be modified based on the findings of the qualitative study.

The presence of malaria infection will be determined using a positive Rapid Diagnostic Test (RDT). Finger or heel prick blood samples (neonates) will be collected. Individuals with fever (axillary temperature ≥ 37.5 °C) and a positive RDT test will receive a treatment dose of artemether–lumefantrine or be referred to a health facility for further care. Participants who test positive for malaria using RDT and have received a full treatment course of an ACT within the 2 weeks preceding the interview will be referred to a health facility if they still experience fever 2 days after completing the last dose of ACT. Cases indicating severe malaria, severe anemia, non-malarial illnesses, or illnesses deemed to require treatment beyond oral anti-malarials will be referred to nearby health facilities. Multiple blood spots samples (usually four) will be obtained by spotting approximately 30 μl of finger-prick blood onto Whatman 3MM filter papers (Maidstone, UK). These blood spots will then be transported to the research offices at the University of Ibadan and Bayero University, Kano, where they will be air dried overnight and stored with silica gel absorbent at room temperature in plastic bags until DNA extraction. The dried blood spots stored in Kano will be transported in cool dry box/container to the University of Ibadan. Polymerase Chain Reaction (PCR) analysis of the malaria samples will be conducted at two well-established laboratories experienced in PCR analysis: Institute of Advanced Medical Research and Training, College of Medicine, University of Ibadan and the Medical Microbiology and Parasitology Department at Ladoke Akintola University of Technology Ogbomosho will conduct all PCR analyses. Since the testing will be pooled across study sites, the participants would likely wait about a month before PCR results can be communicated to them through text messaging.

In the process of selecting households for the study, each ward is divided into census Enumeration Areas (EAs), which will serve as the primary sampling units (PSUs). A household listing procedure will be conducted to determine the number of households and the population in each EA.

##### Initial household and individual-level sample size estimation

Sample size estimation for the survey was conducted using a cluster sampling methodology. Malaria prevalence estimates of children under the age of 5 years from all urban clusters in Oyo and Kano state in the 2018 NDHS (29% and 27% prevalence, respectively) were used to approximate ward-level prevalence estimates in all ages and children under the age of 5 years in Ibadan and Kano, respectively. The expected prevalence values were combined with a design effect ($$Deft$$) of 1.2, a relative standard error (RSE), (a) of 0.05, and an assumed 90% response rate at the household ($${R}_{h})$$ and individual levels $${(R}_{i})$$ to estimate the number of households per study ward as 1741 in Ibadan and 1923 in Kano. The following formula was employed to estimate the sample size for households.

$$n = Deft^{2} \times \frac{1/P - 1}{{\alpha^{2} }}$$

$$n_{i} = \frac{n}{{R_{h} *R_{i} }}$$

where $n$ is the household interview sample size without accounting for non-response, and ${n}_{i}$ is the household interview sample size after adjustment for non-response.

The administration of RDTs is anticipated for a maximum of five individuals per household, depending on the size of the household. Assuming an average household size of 3.9 individuals in Ibadan and 5.7 individuals in Kano, it is estimated that there will be approximately 6111 individuals per ward in Ibadan and 8654 individuals per ward in Kano available for RDT after factoring a decrease in sample size due to an anticipated 90% response rate.

##### Final sample sizes after sample allocation

To allocate the estimated household interview sample sizes of 1741 and 1923 per ward in Ibadan and Kano, respectively, participants in the multistakeholder dialogue and members of the field team will visit each study ward to validate the community maps sketched during the multistakeholder dialogue, map out EAs, and determine whether each EA is a formal settlement, informal settlement or slum using a checklist created from the products of the MSDs. Subsequently, 35 settlements/EAs from each study ward in both Ibadan and Kano will be selected, with the distribution of these EAs proportionate to the ratio of formal settlements, informal settlements, and slum settlements within each ward. By dividing the total household sample size (1741 and 1923 respectively in Ibadan and Kano) by the 35 selected EAs, it is estimated that approximately 50 households (rounded to the nearest whole number) will be interviewed per EA in Ibadan, and 55 households in Kano. Consequently, a total of 1750 households per ward per season in Ibadan and 1925 households per ward per season in Kano will be sampled (Table 5). Considering a 90% response rate, the estimated number of RDTs to be administered per study ward, per season in Ibadan (assuming an average of 3.9 persons per household in Ibadan, 5.7 persons in Kano, and administration of a maximum of 5 RDTs) was decreased to 6143 in Ibadan and 8663 in Kano. In total, across all four wards in Ibadan and for each season, it is expected that 7000 households will be sampled, and 24,572 RDTs will be administered. Similarly, in Kano, for all five wards and each season, 9625 households are expected to be sampled, and 43,315 RDTs will be administered.

**Table 5:** Sample sizes for household interviews and related RDTs

Study site	Total number of wards to be sampled	Household interview sample size per ward and season (total for all wards and each season	RDTs expected to be administered per ward and season (total for all wards and each season)
Ibadan metropolis	4	1750 (7000)	6143 (24,572)
Kano metropolis	5	1925 (9625)	8663 (43,315)

##### Household sampling strategy

Study households in the metropolises of Ibadan and Kano will be selected through random sampling, and interviews will be conducted with the heads of households or their representatives. When there are five or fewer household members, all individuals will be administered RDTs in the household. If there are more than five household members, they will be stratified into five age groups (0–5, 6–10, 11–17, 18–30, and > 30), and one member will be tested from each group. In households with more than five members and missing individuals in a specific age category, an additional household member will be tested from the youngest age category to compensate. Household residents who undergo malaria testing with RDTs will also provide blood spot samples. Research assistants who will receive a 2-day training workshop on survey procedures and a refresher course on sample collection procedures and malaria diagnosis with RDTs, will perform the administration of RDTs and blood spot sample collection using filter paper. Each participant will provide a single blood sample for malaria RDT testing and blotted blood sample on filter paper for PCR analysis. The RDT results will be interpreted according to the manufacturer’s instructions and recorded separately on the participant’s questionnaire. Data, including location information, will be collected through interviewer-administered questionnaires using GIS-enabled Android tablets. This approach will allow for online and real-time access to the collected data.

##### Computation of sampling weights

Due to the non-proportional allocation of the sample size to the different wards and the potential variations in response rates across the wards, sampling weights will be applied. For each individual, the sampling weight will be calculated as the inverse of the first-stage sampling probability of selecting an EA from a specific ward, multiplied by the inverse of the second-stage sampling probability of selecting the household.

#### Health facility surveys

To enhance the mapping of malaria prevalence among children under the age of 5 years in unsampled wards in Ibadan and Kano metro area, surveys will be conducted during both the dry and wet seasons. These surveys will target primigravidae (women experiencing their first pregnancy) who reside in unsampled wards and are seeking antenatal care for the first time at public and private health facilities within those wards. This group of women are the primary focus for the study because their malaria prevalence rates have been found to exhibit the strongest correlation with malaria prevalence in children under the age of five, compared to multigravida [29]. The survey instruments can be found in Additional file 1: pages 79–95. The survey instruments will be updated and refined based on the findings of the qualitative study.

##### Health facility selection

Rather than choosing health facility locations at the ward level, the primary focus for the Ibadan and Kano metro areas will be on the LGAs. This decision was made considering that health facilities usually serve a catchment area that aligns with the LGA. To identify suitable health facilities for the survey, data from the Nigeria District Health Information System (NDHIS2) in 2021, specifically focusing on first-time attendees of antenatal care (ANC) in public and private hospitals, was obtained from the NMEP. Based on this data, one health facility with the highest ANC attendance and within the unsampled wards was purposively selected for each LGA in Ibadan and Kano metro areas. The survey will be conducted monthly for 3 months at each selected health facility, corresponding to both the wet and dry seasons.

##### Sample size estimation and allocation

The sample size for the health facility survey was determined per city, per month. Since there is limited existing literature on malaria prevalence among pregnant women in both Ibadan and Kano metropolises, the estimation considered a positivity proportion of 50% among new ANC attendees, following a similar methodology employed in the NDHS surveys [34]. To adjust for the clustering of pregnant women within a health facility, a design effect of 1.5 was applied, while maintaining a relative standard error (RSE) of 5%. For simplicity, a consistent prevalence rate was assumed throughout the year. Consequently, the sample size was initially determined monthly using the following formula, resulting in an estimated 900 ANC attendees per city.

$$n = Deft^{2} \times \frac{1/P - 1}{{\alpha^{2} }}$$

where $P$ is the assumed malaria prevalence among pregnant women; α is the RSE and $Deft$ is the Design effect.

Since the target population of new ANC attendees per month in Ibadan and Kano was below 900, the estimated sample size was adjusted based on the population size using the average number of ANC attendees between July and September, obtained from routine surveillance data provided by the NMEP. The final sample was calculated using the finite population correction formula indicated below.

$$n^{\prime} = \frac{n}{{1 + \frac{n}{N}}}$$

where $n$ is the estimated sample size; $N$ is the Average of total ANC attendance and $n{\prime}$ is the final sample size estimate.

After accounting for an 85% response rate, the monthly sample size for Ibadan and Kano was increased to 406 and 775 new ANC attendees, respectively. The expected total sample size for 6 months (3 months per season) is 2436 in Ibadan, and 4650 in Kano. Detailed allocation of the sample sizes for Ibadan and Kano metropolitan areas can be found in Tables 6 and 7. The allocation of sample sizes for each health facility was proportionate to its average ANC attendance from July to September 2021 (Tables 6 and 7).

**Table 6:** Sample size allocation for HFS in Ibadan metro

LGA	Health Facility	Average of total new ANC attendees (July–September)	Sample size allocation per month	Sample size for allocation per season (3 months of sampling)
Ibadan North-East	Oluyoro Catholic Hospital	69	50	150
Ibadan North	Adeoyo Maternity Hospital	200	145	435
Ibadan North-West	Naomi Medical Centre	47	34	102
Ibadan South-East	Oranmiyan PHC	131	95	285
Ibadan South-West	Jericho Specialist Hospital	113	82	246
	Total	560	406	1218

**Table 7:** Sample size allocation for HFS in Kano metro

LGA	Health Facility	Average of total new ANC attendees (July–September)	Sample size allocation per month	Sample size for allocation per season (3 months of sampling)
Dala	Dala PHC	431	127	381
Fagge	Rijiyar Lemo MCH Clinic	365	107	321
Gwale	Kabuga PHC	686	202	606
Kano Municipal	Sharada PHC	315	93	279
Nasarawa	Gwagwarwa PHC	538	158	474
Tarauni	Unguwa Uku PHC	300	88	264
	Total	2634	775	2325

##### Sample collection

All new ANC attendees from unsampled wards at the health facility will be interviewed and screened for malaria using RDT until the required number per health facility and month, as shown in Tables 6 and 7, is reached. The data to be collected will be similar to the information gathered in the household cross-sectional surveys. In a designated area/room within the health facility (ensuring confidentiality), Research Assistants who have undergone a 2-day training workshop on survey procedures and a refresher course on blood sample collection and malaria diagnosis using RDT will screen and interview the new ANC attendees. Each pregnant woman will provide a single finger-prick blood sample for the malaria RDT, and for additional confirmation with PCR, the technologist will collect blood spot samples. The RDT results will be interpreted following the manufacturer’s instructions and documented separately on the patient’s questionnaire. Test results will be shared with the health care provider to inform patient management. Similar blood spot analysis protocols as the cross-sectional prevalence surveys will be followed. Data will be collected using GIS-enabled tablets through interviewer-administered questionnaires.

### Longitudinal surveys

To estimate malaria seasonality and related factors, a cohort study will be conducted, following a sample of children aged 0–10 years for 12 months. Data collection will be carried out using survey instruments from Additional file 1: pages 96–117.

Sample size: The sample size per ward was estimated using the OpenEpi open-source sample size calculator for cohort studies [30], accessed at https://www.openepi.com/SampleSize/SSCohort.htm. Based on the methodology described by Fleiss et al.[31], an initial sample size of 228 per ward was estimated. All input parameters used for the sample size calculation are provided in Table 8. Accounting for 20% attrition rate, the minimum sample size was computed as 285 children per ward. Two wards, one with a high proportion of informal settlements and slums and another with a high proportion of formal settlements, will be purposively selected in Ibadan and Kano for the longitudinal survey. Therefore, the total sample size for the two selected study wards in both Ibadan and Kano will be 570 children.

**Table 8:** Parameters used for computing longitudinal study sample size

Parameter	Value (source)
Two-sided significance level	95%
Power	80
Ratio of unexposed to exposed in sample	1.0
Percent of unexposed with outcome (percent of ITN users with malaria)	30.33% (based on malaria incidence estimates from the WHO Observatory [52])
Risk Ratio	0.49 [53]

Participant selection and field methods: Participants will be selected/recruited at the community level. A household listing will be conducted, this list will serve as a sampling frame for randomly selecting eligible households with mother–child pairs. To be eligible for enrollment, it is required that the mother–child pair continue residing in the household for the next year. Within each study ward, two individuals residing there will be recruited and trained to serve as Community Monitors (CMs). Their responsibilities will include identifying eligible study participants, conducting interviews, and administering RDT tests on the study participants. Furthermore, a supervisor will undergo training to oversee the activities of the CMs in each ward, as well as monitor data collection and follow-up processes. Mothers/caregivers of children will be provided with a clear explanation of the study, and those who provide informed consent will be enrolled. Throughout the 1-year follow-up period, participating mothers/caregivers and selected children will receive home visits once a month.

During the initial visit, the CMs will gather information regarding the participant’s family, including basic demographic details and their usual malaria prevention practices. Participants will also be instructed to report to the CMs in their ward if their child under the age of five develops a febrile illness. At each monthly visit, the CMs will administer a questionnaire (refer to Additional file 1 for a sample) to collect information on illnesses that have occurred since the last visit, symptoms experienced, utilization of healthcare facilities, and the use of medications and vector control measures. Additionally, data on travel history will be collected. Anthropometric measurements will be taken, and axillary temperatures and finger or heel prick blood samples (neonates) will be collected for RDT to determine the presence of malaria parasites. Blotted samples on filter paper will be obtained for PCR to confirm cases detected using RDT. PCR analysis protocols and communication of results will follow the same process as the cross-sectional surveys. If a recruited child exhibit fever (axillary temperature ≥ 37.5 °C) and malaria parasitaemia deemed as uncomplicated malaria, they will be administered a treatment dose of artemether–lumefantrine or referred to a health facility. Children who test positive for malaria using RDT and have received a full treatment course of an ACT within the 2 weeks preceding the interview will be referred to a health facility if they still have a fever 2 days after completing the last dose of ACT. Cases indicative of severe malaria, severe anemia, non-malarial illnesses, or illnesses requiring more than oral anti-malarial treatment will be referred to local health facilities. To minimize loss to follow-up, telephone numbers of the mother/caregiver and another close household member who can be contacted to verify their whereabouts will be recorded. During unscheduled visits with the CM, which occur when the child has a suspected case of uncomplicated malaria, similar protocols as in the monthly visits will be followed.

#### Entomological surveys

To gain insights into the larval habitats, indoor and outdoor transmission rates, species composition and dynamics, biting rates, and inoculation rates of *Anopheles* vectors, entomological surveys will be conducted during a 3-month period in the dry season.

##### Selection of the study sites

Considering the expected similarity in vector dynamics among wards with similar environmental conditions, Entomological surveys will be conducted in three wards in Kano and Ibadan, respectiveley, selected based on the outcomes of the clustering process and site visits, as outlined in Table 2. Following the NMEP protocol for entomological surveys, collection sites for both mosquitoes residing indoors and outdoors will be established in each ward. To proceed with the surveys, individuals living in households selected for the study will be engaged and requested to complete informed consent forms (refer to Additional file 1: page 118).

##### Indoor and outdoor mosquito collection with Centers for Disease Control (CDC) light trap

For each site, CDC light traps will be installed both indoors and outdoors within the selected households. The specific details regarding the number of households and the monthly collection frequency can be found in Table 9. The traps will be positioned at a height of 1.5 m near the lower end of an untreated bed net occupied by a sleeping adult. Following the NMEP protocol for routine indoor and outdoor mosquito collection using CDC light traps, operation of the traps will run continuously from 6 p.m. to 6 a.m. for 3 consecutive days each month. Hourly, the cups of the traps will be collected, and the mosquitoes captured will be euthanized using chloroform and carefully transferred into properly labeled cups for identification purposes.

**Table 9:** Number of wards and households for indoor and outdoor mosquito collection and pyrethrum spray catches per season

Month	Dry season—number of study wards	Dry season—number of households per ward for indoor and outdoor mosquito collection	Dry season—number of households per ward for Pyrethrum spray catches
1	3	1	10
2	3	1	10
3	3	8	20

##### Collection of indoor resting mosquitoes using pyrethrum spray catches

At each site, houses will be randomly selected to perform pyrethrum spray catch (PSC) to collect indoor resting mosquitoes. Please refer to Table 9 for the specific details regarding the number of households and the monthly collection frequency per season. The PSC method entails spreading a white cloth across the entire area of the selected household, followed by the application of an insecticide by the Entomologist. Mosquitoes that fall onto the cloth will be carefully collected into labelled petri dishes using forceps. Subsequently, the petri dishes will be securely labelled and transported to the laboratory for identification.

##### Collection of environmental data

During each trapping day, a digital device (Thermo Pro, remote sensor 433 MHz wireless-Model TP-60) will be used to gather data on indoor and outdoor temperature, as well as relative humidity. The collected data will be recorded in hardcopy forms for documentation purposes.

##### Management and identification of collected mosquitoes

Mosquitoes collected in the field (indoor, outdoor, and PSC) will be carefully transported to the laboratory and sorted according to the hourly catch. Female *Anopheles* mosquitoes will be identified using the identification keys developed by Coetzee and Gillies [32] and Gillet [33]. The number of each species of female Anopheles mosquitoes will be recorded, assigned a code, preserved in a silica-filled Eppendorf tube (1.5 ml), and transported to the NMEP-accredited Molecular Biology Laboratory at Osun State University. In the laboratory, the mosquitoes will undergo molecular identification of species, blood meal source, and sporozoite rate using the detailed protocols provided in Additional file 1.

##### Prospection of the breeding sites and duration

Larval sampling will be conducted in all selected wards in Kano and Ibadan metropolis during 3 months in the dry season. Prospective breeding sites will be identified and sampled using standard dippers [34]. When larvae are encountered, they will be carefully transferred to the collection bowl and transported to the laboratory. The procedure for mosquito larval collection at breeding sites is as follows:

1. Larval sampling will be performed between 0700 to 0900 h. Potential breeding sites, including puddles, vehicle tires, septic tanks, gutters, tire tracks, wells, run-offs, and water bodies will be located.
2. The dipper will be gently lowered at an angle of approximately 45° to enable water and nearby larvae to flow into the dipper.
3. The content of the dipper will be emptied into a container and transported to the laboratory. Five dips will be taken per breeding sites.
4. For each sampling operation, the following will be recorded: (a) Geographic location (GPS coordinates), (b) Name of the locality, (c) Type of breeding sites (permanent, semi-permanent or temporary), (d) Source of the water (rain, river, lagoon or man-made), (e) Nature of water collection (puddle, rice-fields, etc.), and (f) Number of larvae per dip.

##### Larval identification and species characterization

The collected larvae will be reared until they develop into adult mosquitoes, which will then be morphologically identified using established protocols for female Anopheles mosquitoes. For further species identification, molecular techniques described in Additional file 1 will be employed.

### Ethics approval and consent to participate

This study has received approval from multiple ethics committees, including Nigeria's National Health Research Ethics Committee (Approval Number: NHREC/01/01/2007-10/10/05/2022), the Health Research Ethics Committees of Oyo State Ministry of Health (Reference number: AD 13/479/44421A) and Kano State Ministry of Health (Approval Number: NHREC/17/03/2018), the University of Ibadan Ethics Committee (Registration number: NHREC/05/01/2008a), Osun State University Ethical and Research Committee (ARIP/UHRC/03), and Northwestern University (IRB ID: STU00217380-MOD0001).

Prior to study enrollment, participants will be requested to provide informed consent, which can be given in written or verbal form. Different informed consent forms are available for each study component, such as qualitative studies, household surveys, health facility surveys, longitudinal surveys, and entomological surveys (refer to Additional file 1 for details). The informed consent forms will be provided in both English and the primary local languages of the study sites (Yoruba and Hausa). Furthermore, during community mobilization, the study information will be presented to the communities, and consent will be sought from the community gatekeepers. The gatekeepers will also be given information on the informed consent process, and sample consent forms will be made available for their review. In the longitudinal study, written informed consent will be required from the parent/guardian of children aged 0–10 years. If the parent/guardian agrees to their child's participation, they will be asked to sign two copies of the written informed consent. One copy will be provided to the parent/guardian, and the other will be retained by the investigator (IA or MA) for the household survey or the health facility worker for the health facility survey. A photocopy of the consent form from the health facility survey will be stored in the investigator’s office. Furthermore, consent will also be obtained directly from the children selected to participate in the study, in addition to parental consent.

### Public involvement

This study is conducted at the request of the NMEP and in partnership with them to generate evidence to inform intervention tailoring in urban areas in the 2026–2030 National Malaria Strategic Plan. Prior to the start of the study, meetings were held with key stakeholders, including the NMEP, to understand their needs and priorities. To ensure that study activities and findings met the needs of the policymakers and the local community, the study protocol and instruments were co-designed in collaboration with NMEP and other key stakeholders including some officials of Ministry of Health in the two States and community members. Additionally, the stakeholders meetings and formative research also added public perspectives to the design and implementation of the study. Community sensitization to inform residents of the study goals and procedures were conducted prior to formative work.

**Fig. 5:**
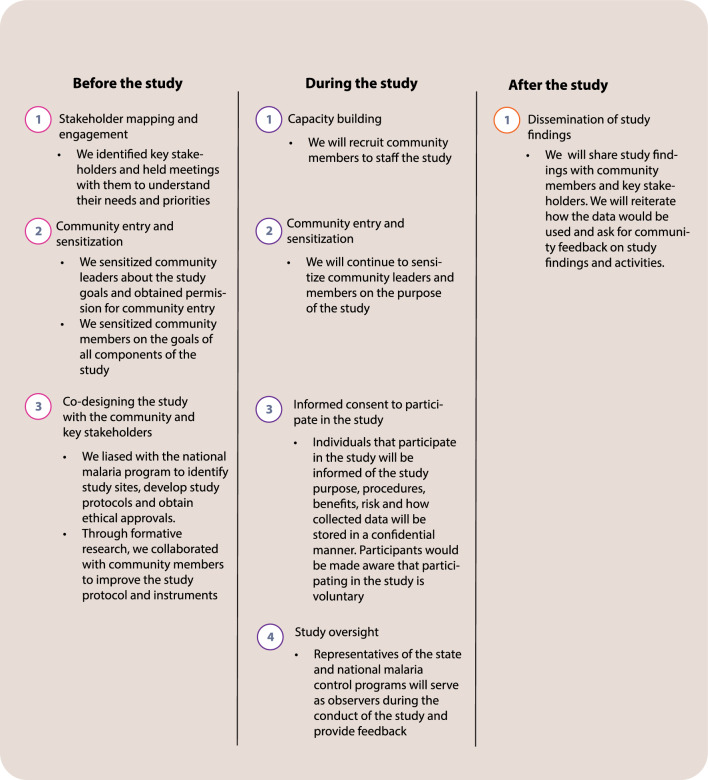
Community engagement plan

During the study, the research capacity of community members will be strengthened by recruiting them as project staff and field assistants. Throughout the study period, community members will be engaged to sensitize them on the study goals and ensure that participants provide informed consent. Plans are in place for representatives of the state and national malaria control programs to serve as observers during the conduct of the study and provide feedback. At the conclusion of the field work, study findings will be shared with community members and key stakeholders including NMEP, Ministry of Health at state level as well as the PHC Coordinators at the LGA level. The feedback of all key stakeholders will be sought on the study findings and activities, including their opinion on any planned interventions. Figure 5 is a diagram of the community engagement plan.

### Data analysis plan

#### Qualitative data

Data processing will begin with the verbatim transcribing of tape recordings of the MSDs, FGDs, KIIs and CIs immediately after the data collection to avoid loss or omission of important details. The findings from the MSDs, FGDs and interviews (KIIs and CIs) conducted in Yoruba and Hausa, will be translated into English, and the data’s quality will be assessed. Project team members will audit and validate all transcribed notes. The validated transcribed notes will be entered into the computer using NVIVO version 12 Pro. The coding process will be driven by an inductive coding approach [35]. Based on the data’s content, parent nodes (primary codes) and child nodes (secondary codes) will be generated. The codes will be linked to their corresponding quotations. Memos will be generated as needed and associated with relevant codes and quotations. The data analyst and experienced qualitative research experts on the project team will meticulously review and critique the generated codes and quotations.

Thematic content analysis will be conducted, and generated themes will be based on the (a) content of the study instrument (b) sample quotes from transcripts; and (c) peer review and reflections contributed by the project team members. Following the step-by-step approach to thematic analysis identified by Nowell et al*.* [36], each verbatim transcript of the MSDs, FGDs and interviews (KIIs and CIs) will be carefully read, examined, and juxtaposed theme by theme to identify relevant texts, repeating words, similar phrases, and divergent opinions. For each theme, common and peculiar trends, as well as similar and divergent opinions, will be noted. The themes will be developed and revised through an iterative process. Furthermore, the explicit and implicit content of the MSDs, FGDs, KIIs, and CIs data will be examined. A summary of the findings will be written, accompanied by relevant verbatim quotes. The findings from the qualitative data will be utilized to inform the development of questionnaires for the cross-sectional and longitudinal studies.

#### Household, health facility and longitudinal surveys

De-identified data from the household and health facility surveys will undergo descriptive analysis to generate preliminary estimates of malaria prevalence during the dry and wet seasons for both sampled and unsampled wards in the Ibadan and Kano metro areas. Smoothed estimates for all wards will be generated using spatial interpolation methods, utilizing information from wards with more data (sampled in the household surveys) to estimate prevalence in areas with limited data points (unsampled wards with malaria test data from the health facility surveys). Furthermore, descriptive analysis will be performed to estimate bed net usage, bed net access, and mobility metrics at the ward level. Additionally, regression models will be employed to investigate the relationship between observed malaria prevalence and factors at both the individual and community levels.

De-identified data from the longitudinal surveys will be subjected to descriptive analysis to determine monthly malaria test positivity rates by RDT specifically among children aged 0–10 years in the Ibadan and Kano metro areas. Regression-based methods for analysing repeated measures will be employed to investigate the relationship between individual and community factors and malaria cases.

#### Entomological data

The data collected through entomological surveys will be analysed to assess the following variables: (1) habitat occupancy, (2) *Anopheles* mosquito species types and their respective habitats in each site during both seasons, (3) larval density, (4) rate of mosquito bites on humans, (5) human blood index, (6) density of mosquitoes resting indoors, and (7) Sporozoite rate. Additional file 1: page 129 provides a comprehensive explanation of the methods used to assess these entomological variables. *Anopheles* mosquito habitats will be georeferenced, and a habitat distribution map will be generated for each study ward.

#### Mathematical modelling

Utilizing the Epidemiological MODeling software (EMOD), ward-level mathematical models will be constructed. EMOD is an agent-based model of malaria transmission that incorporates a susceptible-exposed-infected-recovered (SEIRS) model to track individual disease states [37]. Within the EMOD framework, the calibration of intra-host infections, immune system dynamics, and infection dynamics was performed with field data. As a component of this study, EMOD will be calibrated to all-age prevalence and incidence data obtained from the field study. This calibration aims to capture transmission intensity, considering seasonal variations in vectoral larval habitats in both the Ibadan and Kano metropolis. This study will follow a comparable approach to the one outlined by Ozodiegwu and colleagues [12], while upholding the current general parameters. The model will integrate data on intervention effect sizes, coverages, and distribution schedules. This information will be derived by combining data from existing literature, national surveys, programmatic data, and the data collected during this field study. Simulations will be performed to assess the effects of intervention scenarios outlined by the NMEP on future transmission dynamics to inform urban microstratification strategies.

#### Data confidentiality

All data collected from respondents would be de-identified prior to data analysis. Household level data such as geographic coordinates linking participants to surveys will not be shared publicly and will only be used for data quality assurance and model development. The mathematical models will only produce aggregated projections of ward-level prevalence, incidence, and mortality. All data from the cross-sectional and longitudinal will be stored in REDCap before export into remote encrypted folders/files for data analysis. Only the research team (Principal Investigators—MA, IA, and data analysts who have undergone research ethics training) will have access to the encrypted data.

## Discussion

In urban areas, variations in the epidemiological, entomological, health system, and socio-economic factors influencing malaria morbidity and mortality exist at fine spatial scales, including neighbourhoods, sub-districts, or wards, which are smaller than the usual operational units for intervention targeting, such as states or LGAs. A cross-sectional survey conducted in Accra revealed that communities situated near urban agriculture exhibited a higher prevalence of malaria [38]. Researchers made predictions regarding malaria parasite prevalence at a 10-m grid level for the city of Dar es Salaam, indicating that higher malaria transmission risk was observed along water channels and areas with dense vegetation [39]. Nevertheless, the absence of detailed city maps or plans that delineate agricultural sites and environmental features often hinders malaria control programmes from identifying non-administratively defined units for targeted interventions.

In Nigeria, wards play a crucial role as administratively defined units for political purposes, and they have the potential to effectively capture spatial variations in urban malaria risk. State malaria programmes can readily identify the geographic boundaries of each ward using corresponding polling units and maps, enabling streamlined intervention planning and implementation. Consequently, this served as the primary motivation behind designing the study to capture data at the ward level. Study findings will demonstrate the practicality of using ward demarcations to effectively capture spatial variations in malaria risk.

Informal settlements or slums bear the highest burden of malaria when compared to other urban localities [25]. Existing literature indicates that environmental conditions, human mobility patterns, and limited access to care in informal settlements and slums contribute to local transmission and the introduction and adaptation of parasites in cities [25, 26, 40–44]. It remains uncertain if wards in Nigeria encompass various settlement types. Thus, defining informal settlements and slums would aid in designing sampling strategies that effectively capture heterogeneities in urban malaria risk within and between wards. Despite numerous available definitions of informal settlements and slums [45–48], the definition provided by the United Nations (UN) Human Settlements Programme is widely adopted [45, 47, 49, 50]. As per the UN’s definition, informal settlements “are residential areas where (1) inhabitants have no security of tenure vis-à-vis the land or dwellings they inhabit, with modalities ranging from squatting to informal rental housing, (2) the neighbourhoods usually lack, or are cut off from, basic services and city infrastructure and (3) the housing may not comply with current planning and building regulations and is often situated in geographically and environmentally hazardous areas” [50]. Slums are considered the ‘most deprived and excluded form of informal settlements [49, 50]. However, these definitions may not fully apply to the study communities considering that slums emerge even within government-owned housing projects in Nigeria [51]. Therefore, as an integral aspect of this study’s design, the engagement of community stakeholders and professional experts was imperative in delineating formal and informal settlements as well as slums within the study communities.

Nonetheless, the task of identifying community stakeholders and experts familiar with the geographical locations of distinct settlement types within each study ward presents a challenge. Due to budget constraints, only one multistakeholder dialogue (MSD) could be organized per city. However, a more suitable approach would involve organizing multiple dialogues with various stakeholders to minimize errors in the community maps resulting from the dialogue. To mitigate the chances of errors arising from inaccurate community mapping, members of the field team and participants in the multistakeholder dialogue will physically visit the study sites, validate the maps, and update them as needed, prior to conducting the cross-sectional and longitudinal surveys.

Additional operational and practical challenges during the implementation of this study are anticipated. These challenges may include inaccuracies in locating Enumeration Areas (EAs) within the ward boundaries due to outdated maps, potential refusals to participate in the study, dropouts in the longitudinal study, and concerns regarding data quality that may arise during the survey. To address these issues, several data quality assurance measures have been implemented. These measures include mapping the geographic coordinates of the EAs as they are collected, employing data validation rules, and utilizing skip patterns to safeguard the integrity of the collected data. Furthermore, data collectors will undergo a rigorous training programme plan to ensure their adherence to the prescribed study procedures for data collection. Additionally, this study incorporates plans to raise awareness among community leaders and household members regarding the study's goals, aiming to foster greater participation.

Considering the small-scale nature of data collection, measures to safeguard the confidentiality of participants have been implemented. This includes data de-identification techniques. Information that links to specific households, such as household coordinates, will not be disclosed publicly and will solely be collected to ensure data quality and inform modelling assumptions.

Stratifying urban areas into areas of high and low malaria risk, tailoring interventions accordingly, and mathematical model calibrations to project intervention impact necessitates the availability of context-specific epidemiological, intervention, entomological, socio-economic, climatic, and infrastructural data. Specifically, data needed for mapping malaria risk include information on parasite prevalence, malaria incidence, all-cause and malaria mortality rates; travel histories; occupation; access, coverage, and use of malaria interventions; health services availability; care-seeking patterns; data on vector habitat, species behaviours and insecticide and drug resistance. However, such data is often lacking in control programmes within endemic countries. Recognizing the limited data availability, the WHO suggests that small-scale surveys can be used to establish a baseline level of transmission and identify determinants, while long-term epidemiological surveillance data for microstratification should rely on routine surveillance systems [1]. This study represents an innovative approach to using local communities to guide mapping of potential community characteristics related to transmission in urban areas, and associated instruments can be adapted for use by communities within Nigeria, and in other high-burden countries during such small-scale surveys.

However, when resources for small-scale surveys are not available, data registers at public and private health facilities could be expanded to collect relevant information on determinants, including place of residence, age, travel histories and occupation. This data should be accessible for analysis at both individual and aggregate levels, including health facility, ward, or district levels. This accessibility is crucial to generate evidence for tailoring interventions. Nevertheless, long-term solutions for real-time data access and timely response would require transitioning from paper-based surveys/records to electronic records.

In addition to ad hoc surveys and routine systems, various data sources can contribute to microstratification, including cell-phone call data records, high-resolution satellite imagery, and crowdsourced data from communities. Collaborations between malaria control programmes and mobile phone companies can facilitate access to data on movement patterns, providing insights into their impact on disease transmission. Remote sensing techniques can be employed to gather data on population density, climatic conditions, environmental factors, and larval habitats. Furthermore, community engagement can involve leveraging mobile technology for near-real-time reporting of information related to morbidity (fevers), health-seeking behaviours, and vector behaviours. The cross-sectional study outlined in this write-up encompasses inquiries aimed at gaining a deeper comprehension of engaging community members in reporting malaria cases. Its findings will offer valuable insights for informing future interventions or programs.

## Conclusions

Underpinned by a strong formative research foundation, this project combines epidemiological and entomological study designs to provide understanding of malaria burden and vector transmission dynamics within cities. Data from the field studies will be integrated within a modelling framework to generate information on the impact of reallocation scenarios and/or intervention tailoring scenarios proposed by Nigeria’s NMEP. The studies described in this write-up are illustrative of the types of data and methods for collection needed to inform urban microstratification decisions in malaria-endemic counties.

### Supplementary Information

**Additional file 1. Figure S1:** Overview of methods used to select study locations in Kano metropolis for the household, longitudinal and entomological surveys. Wards visited for ground truthing, and final ward selections are labeled.** Figure S2:** Picture showing two locations in Olopomewa. Roads are tarred and neighborhoods have well-built painted housing infrastructure.** Figure S3:** Picture depicts two locations in Owode. Roads are tarred and there is visible aging of the housing infrastructure.** Figure S4:** Picture showing two locations in Challenge. Roads are untarred and housing types are a combination of modern and old-style gated housing.** Figure S5.** Picture showing two locations in Basorun. Roads are roughly tarred, and housing style is a combination of aging housing and newer cement homes.** Figure S6:** Picture showing two locations in Asanke. Has only major tarred roads but roads are untarred in between houses.** Figure S7:** Picture showing two locations in Agugu. Roads are untarred and have filthy gutters which could serve as breeding sites for mosquitoes.** Figure S8:** Picture showing two locations in Ode Aje. Roads are untarred, housing quality is poor, and drainages are dilapidated or non-existent.** Figure S9:** Picture showing two locations in Bashorun. Roads are tarred and neighborhoods consist of well-built painted housing infrastructure.** Figure S10:** Pictures showing two locations in Dorayi. Housing infrastructure suggests Dorayi has a combination of high and low quality-housing infrastructure.** Figure S11:** Pictures showing two locations in Goron Dutse. Housing infrastructure suggests that Goron Dutse is a rapidly evolving community with ongoing construction. Although houses are not plastered, as shown in the pictures, they are modern buildings. Site reports suggest most areas in the community comprise of low-quality housing.** Figure S12:** Pictures showing two locations in Fagge D2. Housing quality is of medium quality, roads are tarred and there is vehicular access between neighborhoods.** Figure S13:** Pictures showing two locations in Kwachiri. Most buildings are commercial and are used for business.** Figure S14:** Pictures showing two locations in Tudun Wazurchi. Housing is visibly aged and is of medium quality.** Figure S15:** Pictures showing two locations in Sani Mainagge. Housing quality is high, and streets are tarred.** Figure S16:** Pictures showing two locations in Gobirawa ward. Housing type is of medium and poor quality and roads are untarred.** Figure S17:** Pictures showing two locations in Kofar Ruwa depicting the lack of residential structures in this ward.** Figure S18:** Pictures showing two locations in Zango ward, housing quality varies from high to low quality, and drainages are filled with rubbish.** Figure S19:** Pictures showing two locations in Shahuchi. Housing quality varies from medium to poor. Multistakeholder Dialogue Instruments. Focus Group Discussions and Key Informant interview Instruments. Cognitive testing consent form. Cognitive testing discussion guide. Cross-sectional study instruments. Health Facility Survey Instruments. Longitudinal Study Instruments. Entomological survey instruments. Entomological parameters to be collected using study data.

## Data Availability

The Demographic and health survey are publicly available datasets and can be downloaded from https://dhsprogram.com/. Requests for antenatal care attendance data from the Nigeria District Health Information System should be sent directly to the National Malaria Elimination Programme. Access to the National Malaria Strategic Plan 2021–2025 can be provided on request.

## Abbreviations

ANC: Antenatal care
BIC: Bayesian Information Criteria
CDC: Centers for Disease Control
CIs: Cognitive interviews
CM: Community Monitor
EA: Enumeration area
EMOD: Epidemiological MODeling software
FGDs: Focus Group Discussions
HBHI: High Burden to High Impact
HFS: Health Facility Surveys
KIIs: Key Informant Interviews
LGA: Local Government Area
MSDs: Multi-stakeholders’ dialogues
NDHIS2: Nigeria District Health Information System 2
NMEP: Nigerian Malaria Elimination Programme
PCR: Polymerase Chain Reaction
PHC: Primary health care
PSU: Primary sampling unit
RSE: Relative standard error
RDT: Rapid Diagnostic Test
WHO: World Health Organization
